# Association Between the Microsatellite Instability Status and the Efficacy of Postoperative Adjuvant Chemoradiotherapy in Patients With Gastric Cancer

**DOI:** 10.3389/fonc.2019.01452

**Published:** 2020-01-08

**Authors:** Dongfang Dai, Xiaohui Zhao, Xiaoqin Li, Yongqian Shu, Bo Shen, Xiaofeng Chen, Deyu Chen, Deqiang Wang

**Affiliations:** ^1^Department of Medical Oncology, Affiliated Hospital of Jiangsu University, Zhenjiang, China; ^2^Department of Radiation Oncology, Affiliated Hospital of Jiangsu University, Zhenjiang, China; ^3^Department of Pathology, Affiliated Hospital of Jiangsu University, Zhenjiang, China; ^4^Department of Medical Oncology, The First Affiliated Hospital of Nanjing Medical University, Nanjing, China; ^5^Department of Medical Oncology, The Affiliated Cancer Hospital of Nanjing Medical University, Nanjing, China

**Keywords:** microsatellite instability, gastric cancer, chemoradiotherapy, adjuvant therapy, tumor hypoxia

## Abstract

**Purpose:** The effect of microsatellite instability (MSI) on the response to radiotherapy remains unknown. The aim of this study was to investigate the association between the MSI status and the outcomes of gastric cancer (GC) treated by surgical resection with or without postoperative adjuvant chemoradiotherapy.

**Methods:** The records of patients who underwent surgical resection of stage IB–III GC with or without postoperative adjuvant chemoradiotherapy were retrospectively retrieved from the Affiliated Hospital of Jiangsu University (*n* = 89), The Cancer Genome Atlas (*n* = 202), and the Asian Cancer Research Group (*n* = 138). The primary endpoint was overall survival (OS).

**Results:** The MSI status had no significant influence on OS in all cohorts. Compared with surgery alone, adjuvant chemoradiotherapy improved or tended to improve OS of patients with stage III disease, irrespective of the MSI status, in all cohorts. Among patients with stage Ib/II disease, only those with microsatellite stability (MSS) benefited from chemoradiotherapy in terms of OS, whereas those with MSI showed no improvement in OS. A comparison of gene expression profiles between MSI stage Ib/II GC and MSS stage Ib/II GC revealed that MSI correlated with the overexpression of thymidylate synthetase, a marker of fluoropyrimidine resistance. Furthermore, tumor hypoxia scoring for stage Ib/II lesions showed significantly greater hypoxia in MSI tumors than in MSS tumors.

**Conclusions:** The findings of this study suggest that postoperative adjuvant chemoradiotherapy is effective for stage III GC, regardless of the MSI status. However, MSI may predict a poor response to postoperative adjuvant chemoradiotherapy in patients with stage Ib/II GC.

## Introduction

Gastric cancer (GC), which is characterized by epidemiological, biological, and genetic heterogeneity, is a leading cause of cancer-related death worldwide (1). It is associated with a high relapse rate, even after radical gastrectomy. Therefore, postoperative therapies for preventing recurrence have been developed. On the basis of evidence for improved overall survival (OS) and/or disease-free survival (DFS) from randomized phase III clinical trials (2–7), fluoropyrimidine (FU)-based adjuvant chemotherapy or chemoradiotherapy is recommended for patients with resected stage II–III GC and select patients with stage Ib GC who exhibit positive nodes or risk factors for relapse. Several meta-analyses have further confirmed the efficacy of adjuvant chemotherapy or chemoradiotherapy for these patients (8–13).

Although postoperative adjuvant chemotherapy and chemoradiotherapy have resulted in equal improvements in OS in clinical trials (3, 5), chemoradiotherapy may be superior to chemotherapy in terms of local recurrence control. The Adjuvant Chemoradiotherapy in Stomach Tumors (ARTIST) trial found that the addition of radiotherapy to adjuvant chemotherapy improved DFS in patients with node-positive disease and intestinal-type GC (5). Another phase III study of patients with stage III GC treated with R0 gastrectomy and D2 lymph node dissection reported that chemoradiotherapy showed greater beneficial effects than did chemotherapy alone in terms of locoregional recurrence-free survival (3). A recent meta-analysis of seven studies with 1,807 patients also found significant improvements in DFS when radiotherapy was added to adjuvant chemotherapy (14).

In the real world, therapeutic decisions are dependent on individual patient characteristics such as age, performance status, surgical pattern and quality, and preoperative treatment. Currently, both chemotherapy and chemoradiotherapy are considered for patients with resected GC. However, it has been noted that both therapeutic approaches show stage-independent variations in efficacy among patients; therefore, researchers have developed searches for molecular biomarkers that will facilitate the identification of subpopulations that would not benefit from this adjuvant treatment. Nevertheless, most studies aiming to improve patient selection focus on adjuvant chemotherapy rather than chemoradiotherapy.

Comprehensive studies using high-throughput technologies for assessing molecular alterations associated with GC have identified subgroups showing distinct clinical outcomes (15, 16). Notably, microsatellite instability (MSI), which is caused by defects in the DNA mismatch repair (MMR) system, has been associated with a favorable prognosis of GC in several studies (15–18). Meanwhile, some retrospective studies identified a predictive role of MSI in early-stage GC, showing that microsatellite stability (MSS) tumors, but not MSI tumors, can benefit from FU-based adjuvant chemotherapy (17, 19). However, these findings remain inconclusive and controversial (20).

The effect of the MSI status on the response to radiotherapy remains unclear and may be further confounded when chemotherapy and radiotherapy are combined. Accordingly, the aim of the present study was to investigate the association between the MSI status and the outcomes of GC treated by surgical resection with or without postoperative adjuvant chemoradiotherapy.

## Materials and Methods

### Patients

This retrospective study included three patient cohorts. The first one included patients from a prospectively designed database in the Affiliated Hospital of Jiangsu University (AHJU cohort), Zhenjiang, China. The other two cohorts included patients with GC who were selected from The Cancer Genome Atlas (TCGA) and the Asian Cancer Research Group (ACRG) databases. The sample codes for these patients are listed in the Supplementary Data. Relevant data for these patients were retrieved and manually organized as per a previously described protocol (21).

The inclusion criteria for this study were as follows: (1) a history of gastrectomy and FU (5-fluorouracil, capecitabine, or S-1 [tegafur–gimeracil–oteracil potassium capsules])-based chemoradiotherapy [the INT-0116 radiotherapy regimen (7) was a requirement for the AHJU cohort] or simple observation after surgery; (2) a pathological diagnosis of stage Ib–III gastric adenocarcinoma; and (3) no previous history of radiation therapy, chemotherapy, or biological targeted therapy (including preoperative therapy). The American Joint Committee on Cancer criteria was used for clinical and clinicopathological classification and staging.

### Assessment of the MSI Status

Single fluorescent multiplex polymerase chain reaction using genomic DNA extracted from macrodissected cancerous and non-cancerous tissues was performed for determination of the MSI status on the basis of five markers with mononucleotide repeats (15). MSI tumors were defined when samples showed allelic size variations in at least two microsatellites. The remaining samples were considered to represent MSS tumors.

### Pathway Enrichment Analysis and Hypoxia Scoring

mRNA abundance data were freely downloaded from TCGA (https://portal.gdc.cancer.gov/) and the NCBI Gene Expression Omnibus (GEO; GSE62254 for ACRG) and processed as previously described (21). The Kyoto Encyclopedia of Genes and Genomes pathway enrichment analysis was performed using Gene Set Enrichment Analysis (GSEA) software v3.0.

Tumor hypoxia was quantified using Parametric Gene Set Enrichment Analysis (http://www.bioconductor.org/packages/devel/bioc/html/PGSEA.html) based on mRNA-based hypoxia signatures developed by Buffa (22), Winter (23), Ragnum (24), Eustace (25), and Sørensen (26).

### Statistical Analysis

The primary and secondary endpoints were OS and DFS, respectively, which were defined according to a previous study (27). Between-group comparisons were performed using Chi-square tests, Student's *t*-tests, and Mann–Whitney *U*-tests as appropriate. Survival analysis was performed using the Kaplan–Meier method and a log-rank test. Univariate and multivariate Cox proportional hazard models were used for analyses of prognostic factors, with calculation of hazard ratios (HRs) and 95% confidence intervals (CIs). A two-sided *p*-value of <0.05 was considered statistically significant. R (version 3.6.0) and R Bioconductor packages were used for all analyses.

## Results

### Patient Characteristics

The AHJU, TCGA, and ACRG cohorts included 89, 202, and 138 eligible patients, respectively. MSI was correlated (*p* < 0.05; Table 1) with age and the histopathological type in the AHJU cohort; age, sex, and the tumor location in the TCGA cohort; and the tumor location in the ACRG cohort. These findings indicated heterogeneity among the three cohorts.

**Table 1:** Patient characteristics according to MSI status.

**Characteristic**	**AHJU cohort (%)**			TCGA cohort^*^ **(%)**			**ACRG cohort (%)**		
	**MSS**	**MSI**	***P***	**MSS**	**MSI**	***P***	**MSS**	**MSI**	***P***
**Age (years)**									
<65	39 (54.9)	15 (83.3)	0.028	70 (44.3)	10 (23.3)	0.012	49 (50.0)	16 (40.0)	0.286
≥65	32 (45.1)	3 (16.7)		88 (55.7)	33 (76.7)		49 (50.0)	24 (60.0)	
**Sex**									
Female	16 (22.5)	6 (33.3)	0.343	56 (35.4)	24 (54.5)	0.022	25 (25.5)	14 (35.0)	0.261
Male	55 (77.5)	12 (66.7)		102 (64.6)	20 (45.5)		73 (74.5)	26 (65.0)	
**Tumor location**									
Non-antrum	46 (64.8)	8 (44.4)	0.115	105 (69.1)	21 (48.8)	0.014	50 (51.0)	10 (25.0)	0.005
Antrum	25 (35.2)	10 (55.6)		47 (30.9)	22 (51.2)		48 (49.0)	30 (75.0)	
**Histology grade**									
I/II	32 (45.1)	12 (66.7)	0.102	59 (37.8)	13 (30.2)	0.359	50 (51.0)	18 (45.0)	0.521
III	39 (54.9)	6 (33.3)		97 (62.2)	30 (69.8)		48 (49.0)	22 (55.0)	
**Histology type**									
ADC (IT/NOS)	54 (76.1)	18 (100.0)	0.021	120 (76.9)	35 (79.5)	0.713	79 (80.6)	37 (92.5)	0.083
MAC/SRCC/OT	17 (23.9)	0 (0.0)		36 (23.1)	9 (20.5)		19 (19.4)	3 (7.5)	
**TNM stage**									
IB/II	25 (35.2)	7 (38.9)	0.772	88 (55.7)	23 (52.3)	0.686	52 (53.1)	26 (65.0)	0.199
III	46 (64.8)	11 (61.1)		70 (44.3)	21 (47.7)		46 (46.9)	14 (35.0)	
**Adjuvant chemoradiotherapy**									
Untreated	24 (33.8)	6 (33.3)	0.970	118 (74.7)	33 (75.0)	0.966	56 (57.1)	31 (77.5)	0.025
Treated	47 (66.2)	12 (66.7)		40 (25.3)	11 (25.0)		42 (42.9)	9 (22.5)	

* *According to available information*.

*AHJU, Affiliated Hospital of Jiangsu University; TCGA, The Cancer Genome Atlas; ACRG, Asian Cancer Research Group; MSS, microsatellite stability; MSI, microsatellite instability; ADC, adenocarcinoma; IT, intestinal; NOS, not otherwise specified; MAC, mucinous adenocarcinoma; SRCC, signet ring cell carcinoma; OT, others*.

### Association of the MSI Status With the Prognosis

**Figure 1:**
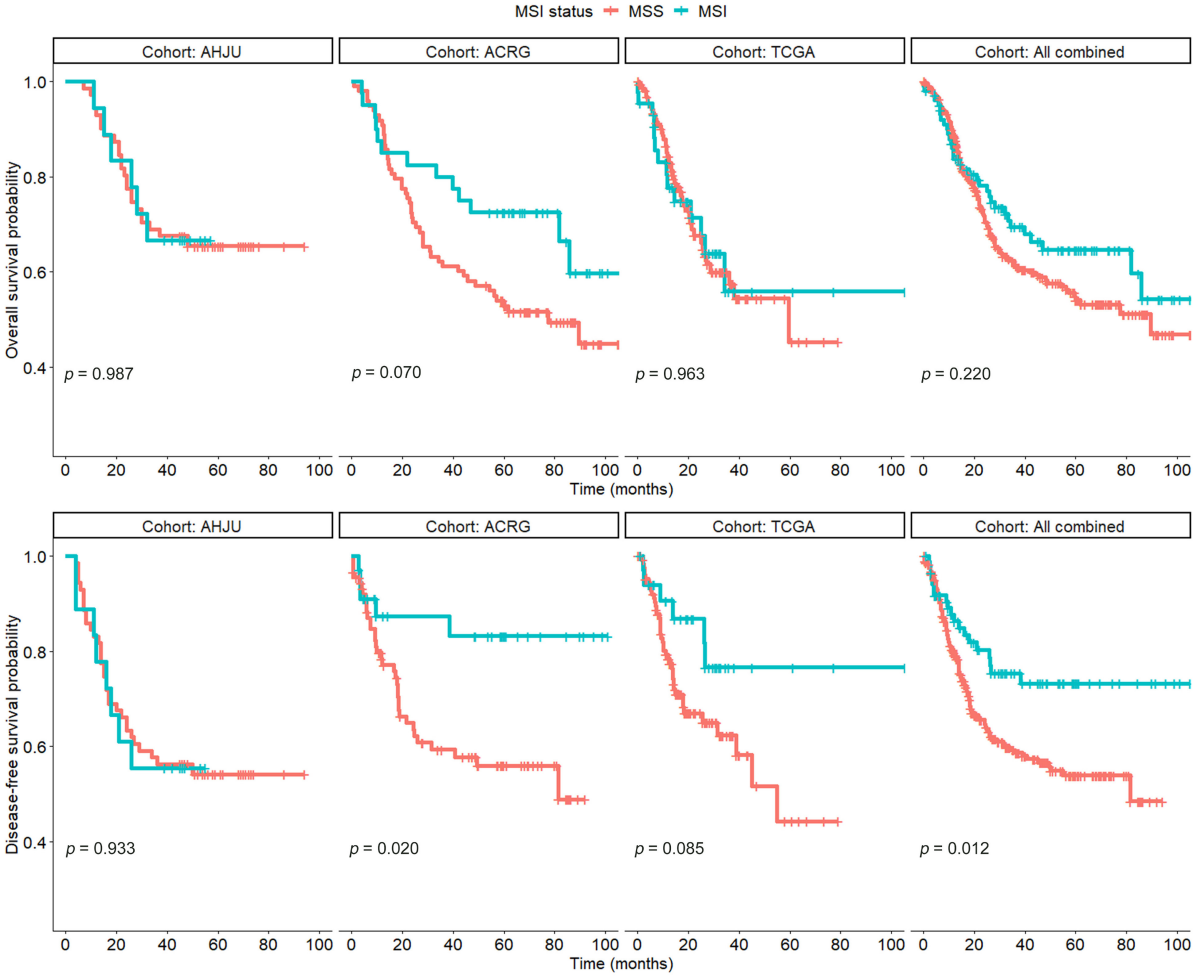
Overall survival and disease-free survival in patients with resected gastric cancer based on microsatellite instability (MSI) status. AHJU, Affiliated Hospital of Jiangsu University; TCGA, The Cancer Genome Atlas; ACRG, Asian Cancer Research Group; MSS, microsatellite stability.

All 429 patients could be analyzed for OS. As shown in Figure 1, there was no significant association between the MSI status and OS in any cohort (*p* > 0.05), although OS tended to be better for patients with MSI GC than for those with MSS GC in the ACRG cohort (*p* = 0.070). In addition, MSI played no prognostic role in terms of OS in a combined cohort including all patients (*p* = 0.220).

A total of 392 patients could be analyzed for DFS, which was significantly better for patients with MSI GC than for those with MSS GC in the ACRG cohort (*p* = 0.020) and the combined cohort (*p* = 0.012). However, there was no significant difference in the AHJU and TCGA cohorts (*p* = 0.933 and 0.085, respectively).

These findings indicated that the prognostic role of MSI in GC needs further clarification.

### Association of the MSI Status With the Efficacy of Postoperative Adjuvant Chemoradiotherapy

**Figure 2:**
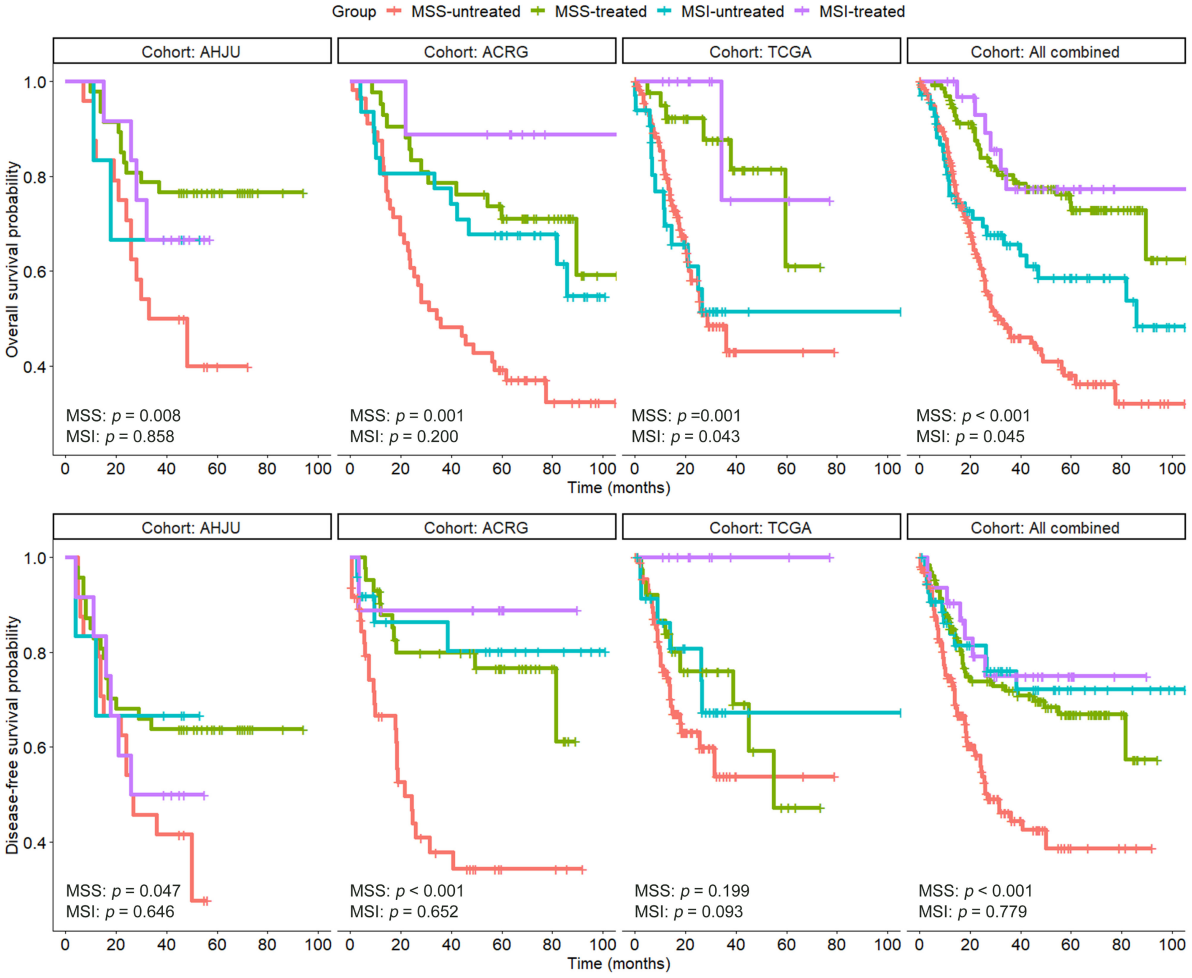
Overall survival and disease-free survival in patients with resected gastric cancer based on microsatellite instability (MSI) and treatment status. AHJU, Affiliated Hospital of Jiangsu University; TCGA, The Cancer Genome Atlas; ACRG, Asian Cancer Research Group; MSS, microsatellite stability.

As shown in Figure 2, compared with surgery alone, adjuvant chemoradiotherapy improved OS of patients with MSS GC in each individual cohort and the combined cohort (*p* < 0.05). Multivariate models showed that chemoradiotherapy was an independent predictor of OS for patients with MSS GC (Table 2) in the AHJU (HR, 0.30; 95% CI: 0.13–0.67; *p* = 0.004), TCGA (HR, 0.29; 95% CI: 0.12–0.71; *p* = 0.006), and ACRG (HR, 0.35; 95% CI: 0.18–0.68; *p* = 0.002) cohorts as well as the combined cohort (HR, 0.28; 95% CI: 0.18–0.44; *p* < 0.001).

**Table 2:** Multivariate analyses of variables associated with overall survival in patients with resected MSS GC.

Variables^*^	**Overall survival**	
	**HR (95% CI)**	***P*-value**
**AHJU cohort**		
Chemoradiotherapy (treated vs. untreated)	0.30 (0.13–0.67)	0.004
TNM stage (III vs. IB/II)	3.04 (1.12–8.24)	0.029
**TCGA cohort**		
Chemoradiotherapy (treated vs. untreated)	0.29 (0.12–0.71)	0.006
Histology grade (III vs. I/II)	2.46 (1.22–4.96)	0.012
**ACRG cohort**		
Chemoradiotherapy (treated vs. untreated)	0.35 (0.18–0.68)	0.002
Age (≥65 vs. <65 years)	1.64 (0.89–3.01)	0.110
TNM stage (III vs. IB/II)	2.33 (1.30–4.18)	0.005
**All patients**		
Chemoradiotherapy (treated vs. untreated)	0.28 (0.18–0.44)	<0.001
Age (≥65 vs. <65 years)	1.31 (0.90–1.91)	0.162
Histology grade (III vs. I/II)	1.36 (0.94–1.97)	0.107
TNM stage (III vs. IB/II)	2.14 (1.47–3.12)	<0.001

* *Variables were adopted for their prognostic significance by univariate analysis*.

*MSS, microsatellite stability; GC, gastric cancer; HR, hazard ratio; CI, confidence interval; AHJU, Affiliated Hospital of Jiangsu University; TCGA, The Cancer Genome Atlas; ACRG, Asian Cancer Research Group*.

DFS of patients with MSS GC was significantly improved by adjuvant chemoradiotherapy in the AHJU and ACRG cohorts and the combined cohort (*p* < 0.001 for all). However, there was no significant improvement in DFS of patients with MSS GC in the TCGA cohort (*p* = 0.199), which may be limited by missing DFS data for 17.1% patients (27/158).

Interestingly, adjuvant chemoradiotherapy significantly improved OS of patients with MSI GC in the TCGA cohort (*p* = 0.043), and a trend for this beneficial effect was also observed in the ACRG cohort (*p* = 0.200). A similar result was observed for the combined cohort (*p* = 0.045). These findings suggest that postoperative adjuvant chemoradiotherapy may be effective for select patients with MSI GC.

### Association of the MSI Status With the Efficacy of Postoperative Adjuvant Chemoradiotherapy by TNM Stage

**Figure 3:**
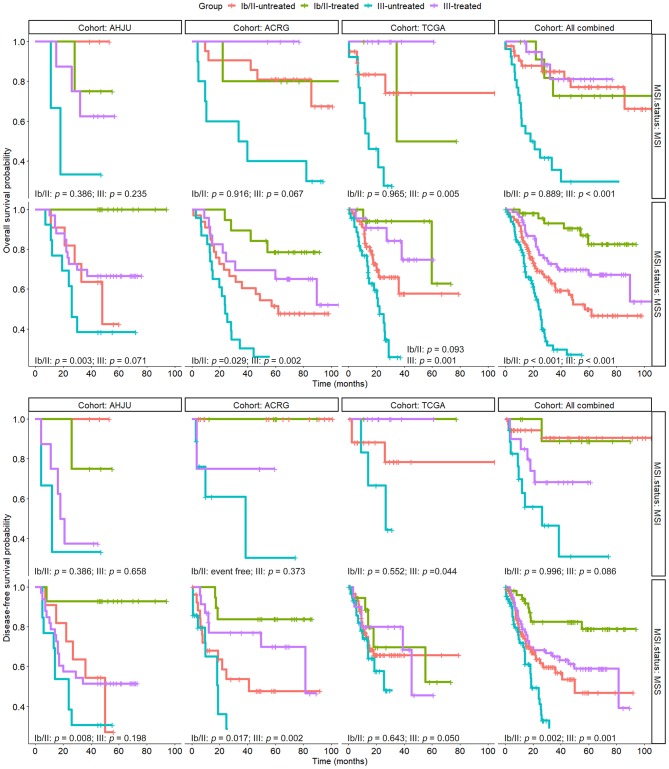
Overall survival and disease-free survival in patients with resected gastric cancer based on treatment status and TNM stage, which was further stratified by microsatellite instability (MSI) status. AHJU, Affiliated Hospital of Jiangsu University; TCGA, The Cancer Genome Atlas; ACRG, Asian Cancer Research Group; MSS, microsatellite stability.

To determine the subgroups of patients with MSI GC that could benefit from adjuvant chemoradiotherapy, we conducted stratified analyses for the association of MSI with the efficacy of chemoradiotherapy according to the TNM stage. As shown in Figure 3, patients with MSS GC benefited or tended to benefit from chemoradiotherapy in terms of OS; this effect was observed for both stage Ib/II and stage III lesions. However, among the patients with MSI GC, beneficial effects of chemoradiotherapy on OS were observed only for those with stage III lesions. These results were further confirmed in the combined cohort. The results of stratified analyses based on the TNM stage were more heterogeneous for DFS, although they were similar to the results for OS in the combined cohort.

### Results of GSEA and Tumor Hypoxia Scoring for Stage Ib/II GC

**Figure 4:**
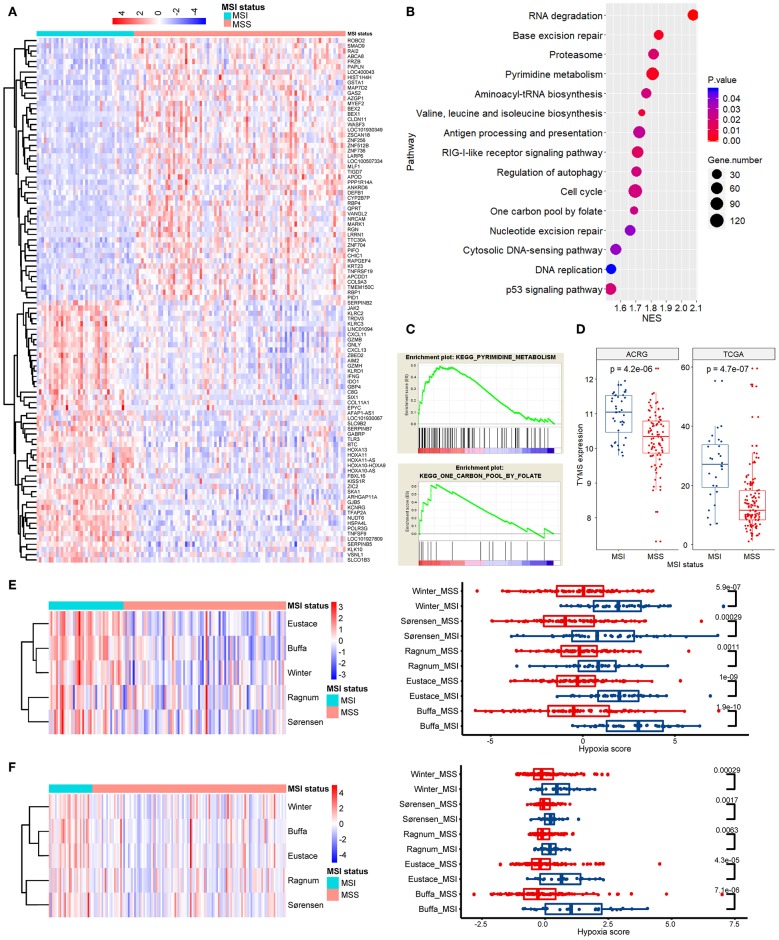
Pathway enrichment analysis and tumor hypoxia scoring. **(A)** A clustered heat map of the differentially expressed genes between MSI and MSS GC of stage Ib/II in ACRG. **(B)** The most significantly enriched signaling pathways in GSEA. **(C)** Enrichment plots for pathways of pyrimidine metabolism and one carbon pool by folate. **(D)** The mRNA expression of thymidylate synthetase (*TYMS*) in stage Ib/II GC is significantly higher in the MSI subtype than that in the MSS subtype in both the TCGA and ACRG cohorts. **(E,F)** The differential hypoxia scores in ACRG **(E)** and TCGA **(F)** between MSI and MSS GC of stage Ib/II shown by clustered heat maps and direct comparisons in each individual signature (authors are shown). ACRG, Asian Cancer Research Group; TCGA, The Cancer Genome Atlas; MSI, microsatellite instability; MSS, microsatellite stability; GSEA, Gene Set Enrichment Analysis; NSE, normalized enrichment score.

The above mentioned results indicated variations in the efficacy of chemoradiotherapy between MSI stage Ib/II GC and MSS stage Ib/II GC. Therefore, we compared the gene expression profiles between these subgroups in the ACRG cohort (Figure 4A). GSEA revealed that the most significantly enriched pathways in MSI GC were involved in DNA repair, nucleotide metabolism, cell cycles, and DNA replication (Figure 4B). In particular, pathways of pyrimidine metabolism and one carbon pool by folate, which are all associated with FU metabolism and resistance, were significantly enriched (Figures 4B,C). We then considered thymidylate synthetase (TS, *TYMS*), an indispensable key enzyme in folate and pyrimidine metabolisms and the target of FU (28). As shown in Figure 4D, *TYMS* mRNA expression was significantly higher in MSI stage Ib/II GC than in MSS stage Ib/II GC in the ACRG cohort, which was further verified in the TCGA cohort (*p* < 0.001 for both).

Hypoxia renders tumors resistant to radiotherapy and chemotherapy (29). Thus, we quantified and compared tumor hypoxia between MSI stage Ib/II GC and MSS stage Ib/II GC. For all hypoxia signatures, MSI tumors showed significantly greater hypoxia than did MSS tumors in both the ACRG (Figure 4E) and TCGA (Figure 4F) cohorts (*p* < 0.05).

## Discussion

For a long time, GC has been suboptimally treated because of the application of a common uniform therapeutic strategy, irrespective of the disease subtype. Recent progress in deciphering the genomic landscape of GC has contributed to the development of novel therapeutic strategies by molecular classification (15, 16). In the present study, we evaluated the association between the MSI status in resected GC and the efficacy of postoperative adjuvant chemoradiotherapy. To our knowledge, this association has not been reported earlier.

The most novel finding in this study was that postoperative adjuvant chemoradiotherapy may have a beneficial effect in patients with MSI stage III GC. Interestingly, a detrimental effect of adjuvant chemotherapy was previously detected for this subgroup (30). However, that study was retrospective and enrolled only 50 patients with MSI stage III GC, including only nine controls; this indicated potential bias. Similarly, in a *post hoc* analysis of the results of the Adjuvant Capecitabine and Oxaliplatin for Gastric Cancer After D2 Gastrectomy (CLASSIC) trial, only 16 patients with stage III disease were included from a total of 40 patients with MSI GC (17). The findings revealed no benefit of adjuvant chemotherapy for MSI GC, with no available results for stage III lesions. Clearly, it is difficult to conduct a sufficiently large study for MSI GC, let alone a study for MSI stage III GC, because the reported prevalence of MSI GC ranges from only 6.6 to 22.7% (15–19, 30–32). In our study, the prevalence of MSI GC was 20.2, 21.8, and 29.0% in the AHJU, TCGA and ACRG cohorts, respectively. However, despite similar sample size limitations, we observed a significant benefit of postoperative adjuvant chemoradiotherapy in patients with MSI stage III GC. This finding suggests a difference in efficacy between chemoradiotherapy and chemotherapy in this subgroup and needs further investigations.

With regard to the effect of the MSI status on the prognosis of GC, we found population heterogeneity among the three cohorts included in our study. Although MSI GC has shown a favorable prognosis in several studies (15–18), it has shown a prognosis similar to that of MSS GC in other studies (19, 30). Interestingly, a prognostic effect of MSI may exist in patients who receive only surgery, and it could be attenuated by the administration of adjuvant chemotherapy (30). This finding was also observed in the present study, particularly in the combined cohort (Figure 2). Taken together, the prognostic role of the MSI status in early-stage GC remains conflicting.

The role of MSI as a biomarker is thought to be mediated by its effect on antitumor immunity (33), which may be attenuated by the immunosuppressive effects of chemotherapy. However, chemotherapy and/or radiotherapy also modify the immune contexture by eliminating immunosuppressive cells or stimulating the immune system (34). It is believed that the ultimate outcome of a treatment for cancer is determined by the equilibrium between its immunostimulatory and immunosuppressive effects (35). In the present study, we found differences in the efficacy of adjuvant chemoradiotherapy between MSI stage III GC and MSI stage Ib/II GC, which indicates that the abovementioned equilibrium differs between these two subgroups.

We also revealed novel potential mechanisms underlying the differential effects of chemoradiotherapy for MSI stage Ib/II GC and MSS stage Ib/II GC. Compared with MSS tumors, MSI tumors exhibited increased expression of genes enriched in specific pathways that may induce resistance to therapy. In addition, tumor hypoxia was significantly greater in MSI stage Ib/II GC than in MSS stage Ib/II GC. Tumor hypoxia plays a central role in the tumor's resistance to therapy (29). Several mRNA-based tumor hypoxic signatures have been shown to predict clinical outcomes in many cancers, independent of existing clinical markers (22–26). To our knowledge, this is the first study to determine differences in tumor hypoxia according to the MSI status by using these well-established signatures.

Limitations of this study primarily include the retrospective design, heterogeneous population, and limited number of patients with MSI GC. However, we evaluated three independent cohorts from different areas and observed a consistent association between the MSI status and the efficacy of postoperative adjuvant chemoradiotherapy in patients with resected GC. This indicates the robustness of our findings, although further prospective validations with well-designed, larger samples are warranted.

In conclusion, the results of this study suggest that postoperative adjuvant chemoradiotherapy has beneficial effects on MSI stage III GC and MSS GC of all stages. However, patients with MSI stage Ib/II GC may respond poorly to adjuvant chemoradiotherapy after resection. These findings are expected to contribute to an improvement in the clinical outcomes of resected GC by improving patient selection for postoperative adjuvant therapy.

## Data Availability Statement

The data sets in this study are available from the corresponding author on reasonable request.

## Ethics Statement

The studies involving human participants were reviewed and approved by Affiliated Hospital of Jiangsu University. The patients/participants provided their written informed consent to participate in this study. All procedures followed were in accordance with the ethical standards of the responsible committee on human experimentation (institutional and national) and with the Helsinki Declaration of 1964 and later versions.

## Author Contributions

DD, XZ, XL, YS, BS, XC, DC, and DW were involved in data interpretation and statistical analysis. DD, XZ, and DW were involved in the design of the study and preparation of the manuscript. All authors reviewed and approved the final manuscript.

### Conflict of Interest

The authors declare that the research was conducted in the absence of any commercial or financial relationships that could be construed as a potential conflict of interest.
